# The Relationship between Anxiety and the Social Judgements of Approachability And Trustworthiness

**DOI:** 10.1371/journal.pone.0076825

**Published:** 2013-10-02

**Authors:** Megan L. Willis, Helen F. Dodd, Romina Palermo

**Affiliations:** 1 School of Psychology, Australian Catholic University, Sydney, New South Whales, Australia; 2 Department of Cognitive Science, Macquarie University, Sydney, New South Whales, Australia; 3 School of Psychology and Clinical Language Sciences, University of Reading, Reading, United Kingdom; 4 School of Psychology, and ARC Centre for Cognition and its Disorders (CCD), University of Western Australia, Crawley, Western Australia, Australia; George Mason University/Krasnow Institute for Advanced Study, United States of America

## Abstract

The aim of the current study was to examine the relationship between individual differences in anxiety and the social judgements of trustworthiness and approachability. We assessed levels of state and trait anxiety in eighty-two participants who rated the trustworthiness and approachability of a series of unexpressive faces. Higher levels of trait anxiety (controlling for age, sex and state anxiety) were associated with the judgement of faces as less trustworthy. In contrast, there was no significant association between trait anxiety and judgements of approachability. These findings indicate that trait anxiety is a significant predictor of trustworthiness evaluations and illustrate the importance of considering the role of individual differences in the evaluation of trustworthiness. We propose that trait anxiety may be an important variable to control for in future studies assessing the cognitive and neural mechanisms underlying trustworthiness. This is likely to be particularly important for studies involving clinical populations who often experience atypical levels of anxiety.

## Introduction

In our daily lives we frequently make judgements about other individuals that influence our willingness to socially engage with them. We often rely on information from an individual's facial appearance to guide these judgements. Given this, there has been considerable interest in elucidating the cognitive and neural mechanisms that underlie the ability to make social judgements from an individual's facial appearance [1], [2], [3].

The social judgements of trustworthiness and approachability have been of particular interest to researchers [3], [4]. Individual faces vary in both their perceived approachability and trustworthiness [3]. An impaired capacity to make these social judgements has been observed in individuals within a number of clinical populations, including those with bilateral amygdala lesions, autism and Williams syndrome [4], [5], [6], implicating the amygdala in this ability [4], [7], [8]. The involvement of the amygdala in the process of making social judgements is thought to stem from its role in threat detection, the assessment of which is thought to be central to the capacity to make appropriate social judgements [9], [10].

Recent evidence has shown that amygdala responses to faces are affected by individual differences in anxiety, with individuals high in anxiety (both state and trait) showing elevated amygdala reactivity to faces depicting threat, in the form of angry and fearful faces [11]. Behavioural evidence has also demonstrated a relationship between individual differences in anxiety and the perception of threat. For instance, higher levels of trait anxiety are associated with the perception of greater hostility from faces [12], as well as enhanced attention to potential threat more generally [13]. Thus, individual differences in anxiety may also be associated with the precise social judgements ascribed to faces, particularly given the importance of the amygdala and threat assessment in making these judgements.

Initial evidence in support of this assertion has been found in individuals with clinical levels of social anxiety. Individuals with heightened levels of social anxiety judge happy faces as less approachable than healthy controls [14]. Social anxiety disorder, or social phobia as it is also known, is a disorder characterised by fear and avoidance of social situations, and is distinct from other anxiety disorders, such as generalised anxiety disorder. State anxiety is a transient emotional response to a particular event, whereas trait anxiety refers to an individual's relatively stable tendency to perceive and respond to situations with elevations in his or her state anxiety. [15].

Despite evidence implicating heightened levels of state and trait anxiety with abnormalities in behavioural and neural responses to faces, the relationship between state and trait anxiety and the social judgements of approachability and trustworthiness has been neglected to date. Investigating this relationship is important, as our understanding of the factors that influence these two social judgements has been centred on their cognitive and neural bases [16]. The contribution of individual differences has received scarce attention in contrast. Not only is understanding the potential contribution of individual differences important for our understanding of the factors that determine these judgements, but it may also have important implications for studies examining social judgements in special populations. Some of the clinical populations that display abnormal social judgements have also been reported to have atypical levels of anxiety [17]–[19]. Research examining social judgements in these clinical populations generally compares affected individuals with normal controls or individuals with different diagnoses [20]. However, if anxiety levels differ between the population of interest and the comparison group, then group differences could be a function of anxiety rather than diagnosis.

The primary aim of the current study was to address this gap. We sought to establish if there is a relationship between state and trait anxiety and the social judgements of approachability and trustworthiness. Here, we opted to focus on social judgements assigned to neutral faces, as evidence for impaired social judgements in clinical populations has largely been observed in studies utilising neutral face stimuli [4], [5], [6], as opposed to discrete emotional categories. Many studies examining the mechanisms involved in making social judgements have investigated either approachability or trustworthiness independently [2], [21], [22], [23]. There are common factors that determine these two social judgements, as indicated by the existence of a moderate to strong correlation between the two social judgements [4], [10]. However, there may also be factors that have distinct effects on the approachability and trustworthiness judgements ascribed to faces. When the two social judgements were assessed in individuals with autism, individuals were observed to have deficits in trustworthiness judgements, but not approachability judgements [5]. A study conducted by Willis et al. [10] also indicated that there might be important differences between the two judgements. They found that emotional expression exerted a stronger effect for judgements of approachability than trustworthiness. Moreover, the effect of eye gaze on the two social judgements was found to be divergent, with faces displaying averted eye gaze considered less trustworthy than those with direct eye gaze. In contrast, no such difference was observed for approachability ratings. We assessed both judgements in this study, as this allowed us to compare whether similar relationships with anxiety are evident.

## Method

### Ethics Statement

This research was approved by Macquarie University's Human Research Ethics Committee (HREC). All participants provided written informed consent to participate in the study.

### Participants

Eighty-two undergraduate students (60 female) whose ages ranged from 18 to 51 (*M* = 24.74, *SD* = 9.46) participated in the study for course credit. Most participants were Caucasian (86.6%), with the remainder Asian (8.5%), African (1.2%), Pacific Islander (1.2%) and Mixed Race (2.4%).

### Stimuli

Photographs of 100 Caucasian (50 female) faces each displaying a neutral pose were sourced from the Karolinska Directed Emotional Faces (KDEF) database [24] and the Radboud Faces Database [25]. The faces (256 grey levels, 72 ppi) were scaled to be the same size, covering a visual angle of approximately 5.2°×7.6°, at a viewing distance of approximately 60 cm on a 17-inch monitor (screen size, 1024×768 pixels). Stimulus presentation was controlled using Superlab (Cedrus Corp.) on Dell OptiPlex GX745 computers.

### Social Judgement Tasks

#### Approachability

We used an approachability task that has been used in previous research [10], [21], [26]. In this task, participants are asked to imagine being in a situation where they are on a crowded street on their way to meet a friend. They are asked to pretend that they are lost and in a hurry and need to ask someone for directions. For each face, participants are asked to imagine seeing the face in the crowd and to indicate the degree to which they agree with the following statement “I would approach this person to ask for directions.”

#### Trustworthiness

The trustworthiness task has also been used previously [10]. Participants are asked to indicate whether they would trust a stranger with their camera. Participants are told to imagine being on a crowded street while on holiday. They are asked to pretend that they have been taking photographs of a famous monument, when a stranger offers to take a photograph of them in front of the monument with their camera. For each face, they are asked to indicate the extent to which they agree with the following statement “I would trust this person with my camera.”

In both the approachability and trustworthiness tasks, the faces were presented one at a time on a white background, in a randomised order. Responses were made on a 9-point likert scale from −4 (strongly disagree) to +4 (strongly agree). The face, statement and scale remained on the screen until a response was made. Participants were asked to use the full range of the scale when completing the task. The approachability and trustworthiness tasks were completed in a counterbalanced order between participants.

### State and Trait Anxiety Measure

After completing the social judgement tasks, we assessed both state and trait anxiety using the Spielberger State-Trait Anxiety Inventory (STAI) [15]. The STAI is the most widely used measure of state and trait anxiety. It assesses state anxiety with 20 statements that participants evaluate with respect to how they feel “right now, at this very moment”. Whereas trait anxiety is assessed with 20 statements that participants evaluate with reference to how they “generally” feel. Responses are made on a four-point likert scale, ranging from Not At All (1) to Very Much So (4). Both state and trait scales have excellent internal reliability with Cronbach's α>.90 in normative samples. In the current sample, internal reliability was also excellent for both state anxiety (Cronbach's α = .91) and trait anxiety (Cronbach's α = .93) scales.

### Statistical Analyses

We first performed Pearson's correlations to examine the nature of the relationship between the variables of interest. We then performed multiple regressions assessing state and trait anxiety as predictors of the outcome variables of approachability ratings and trustworthiness ratings. Age and sex were also included as covariates in the regression models to ensure that any significant relationship emerging between anxiety level and either social judgement could not be attributable to individual differences in age and sex. This was considered important, as both age and sex have been associated with levels of anxiety and/or social judgements in previous studies [12], [21], [27], [28].

Before performing the analyses, the assumptions of linearity, independent errors and homoscedasticity were checked and satisfied. Inspection of Mahalanobis distances indicated that there were no significant outliers in the sample. Collinearity diagnostics were inspected and confirmed there was no evidence of multicollinearity. The assumption of normality was violated, however given that large samples (e.g., *n*>30) are assumed to come from a normal sampling distribution this was not considered problematic [29].

## Results

**Figure 1:**
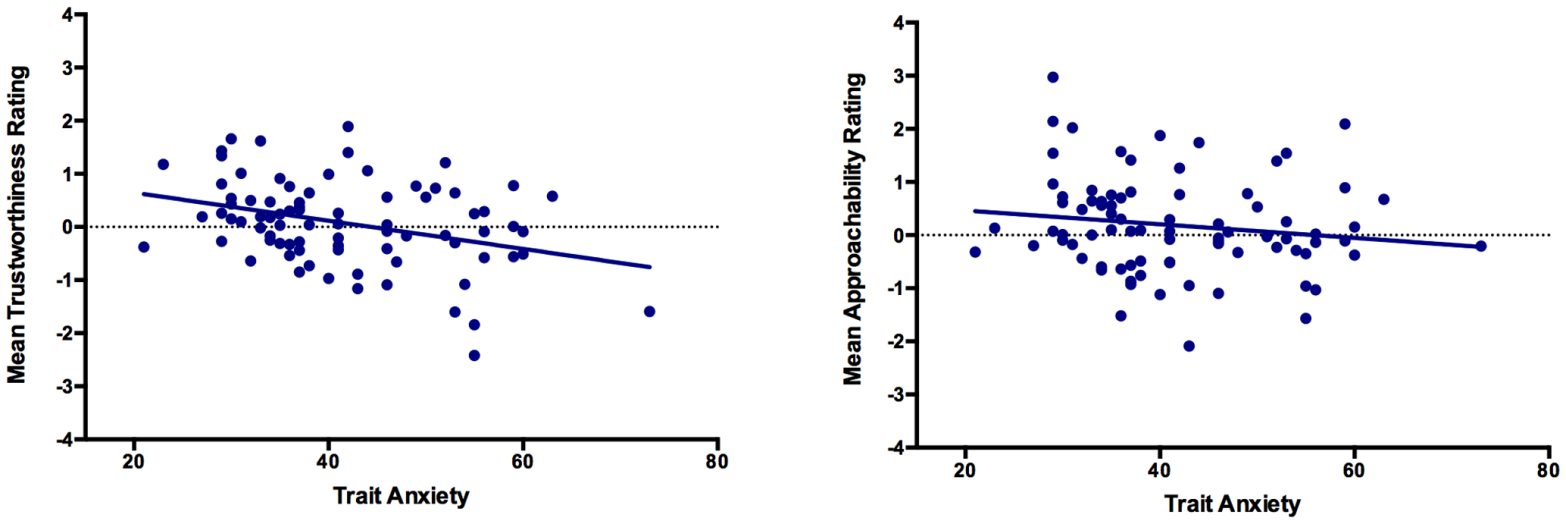
Scatterplots showing the significant negative correlation between mean trustworthiness rating and trait anxiety (left) and the non-significant correlation between mean approachability rating and trait anxiety (right).

Pearson's correlations are displayed in Table 1, along with the mean and standard deviation for each variable. Consistent with previous research [10], approachability and trustworthiness ratings were significantly correlated, as participants who rated faces as more approachable tended to rate faces as more trustworthy. Of particular interest were significant correlations that emerged between trustworthiness judgements and both state and trait anxiety. As shown in Figure 1, participants with higher anxiety levels tended to rate faces as less trustworthy. In contrast, no significant relationship emerged between approachability judgements and state anxiety or trait anxiety (see Figure 1).

**Table 1:** Descriptive Statistics and Zero-order Correlations Between All Variables.

Variable	1	2	3	4	5	*M*	*SD*
1. Approach						.18	.91
2. Trust	.65**					.07	.80
3. State Anxiety	−.21	−.24*				37.90	9.83
4. Trait Anxiety	−.15	−.35**	.69**			41.59	10.55
5. Sex	.21	.15	−.12	−.11		1.27	.45
6. Age	.20	.18	−.20	−.23*	.02	24.74	9.46

*p<.05, two-tailed.

**p<.005, two-tailed.

Tables 2 and 3 show the unstandardized regression coefficients (*B*), the standard error (*SE*), the standardized regression coefficients (*β*), and zero-order and partial correlations for the four variables entered into the regression model for trustworthiness (Table 2) and approachability (Table 3). The regression model predicting trustworthiness ratings from the four variables was significant, *F*(4,77) = 3.08, *p* = .021, and accounted for 14% of the variability in trustworthiness judgements. As Table 2 shows, trait anxiety was the only variable that was a significant predictor of trustworthiness judgements. Hierarchical regression analyses revealed that trait anxiety accounted for unique variance in trustworthiness judgements above and beyond the demographic variables of age and sex, *R^2^* change  = .09, *p* = .005, whereas state anxiety did not, *R^2^* change  = .04, *p* = .077. Trait anxiety also explained a significant proportion of variance beyond that of state anxiety, age and sex, *R^2^* change  = .06, *p* = .027.

**Table 2:** Regression Analyses Predicting Trustworthiness Judgements From Sex, Age, State Anxiety and Trait Anxiety.

Predictors	*B*	*SE*	*β*	*t*	Correlation	
					Zero-order	Partial
Constant	.647	.569				
Sex	.202	.193	.112	1.05	.15	.12
Age	.006	.009	.075	.69	.15	.08
State Anxiety	.002	.012	.022	.15	−.24*	.02
Trait Anxiety	−.025	.011	−.333	−2.25*	−.35**	−.25*

*p<.05, two-tailed.

**p<.005, two-tailed.

**Table 3:** Regression Analyses Predicting Approachability Judgements From Sex, Age, State Anxiety and Trait Anxiety.

Predictors	*B*	*SE*	*β*	*t*	Correlation	
					Zero-order	Partial
Constant	−.189	.658				
Sex	.384	.223	.188	1.73	.21	.19
Age	.016	.011	.165	1.48	.20	.17
State Anxiety	−.017	.014	−.18	−1.19	−.21	−.13
Trait Anxiety	.003	.013	.033	.22	−.15	.02

*p<.05, two-tailed.

The regression model predicting approachability judgements failed to reach significance, *F*(4,77) = 2.23, *p* = .074. The model accounted for 10.4% of the variability in approachability judgements, however none of the four predictors were significant.

Inspection of partial correlations in Tables 2 and 3 revealed that only the correlation between trait anxiety and trustworthiness remained significant after controlling for all other variables. To determine if trait anxiety was a significantly stronger predictor of trustworthiness ratings than state anxiety, we statistically compared the two partial correlations using William's T_2_ statistic. William's T_2_ statistic is the recommended test for assessing the equality of two dependent correlations [30]. The test statistic indicates whether there is a significant difference between the strength of two correlations obtained from the same individuals. The test revealed that the partial correlation between trait anxiety and trustworthiness judgements was significantly stronger than that observed between state anxiety and trustworthiness judgements, *t*(79) = 3.16, *p*<.005.

Also of interest was whether the partial correlation between trait anxiety and trustworthiness judgements was significantly stronger than the partial correlation between approachability judgements and trait anxiety. Calculation of William's T_2_ statistic revealed that the partial correlation between trait anxiety and trustworthiness was significantly stronger than that observed between trait anxiety and approachability judgements, *t*(79) = 3.08, *p*<.005. Thus, suggesting that trait anxiety is a significantly greater predictor of trustworthiness judgements than approachability judgements.

## Discussion

The aim of the current study was to explore whether individual differences in state and trait anxiety are associated with approachability and trustworthiness judgements. We were also interested in whether any relationships would be equivalent for trustworthiness and approachability judgements. A significant relationship was found between trait anxiety and trustworthiness after controlling for age, sex and state anxiety. In contrast, state anxiety was no longer a significant predictor after controlling for other variables. This suggests that trustworthiness judgements are more closely related to an underlying anxious disposition than how the participants felt at the point when they completed the measures. No significant relationship emerged between state or trait anxiety and approachability judgements. Of all four predictors included in the regression model, only trait anxiety was a significant predictor of trustworthiness ratings. For approachability in contrast, none of the predictors accounted for a significant degree of variation in approachability ratings.

Interestingly, trustworthiness judgements had a significantly stronger relationship with trait anxiety than that seen for approachability judgements. While approachability and trustworthiness judgements are strongly correlated, the current findings illustrate that they are clearly distinct constructs that are differentially influenced by levels of trait anxiety. One possible explanation for the relationship between trait anxiety and trustworthiness judgements is that the judgement made in the trustworthiness task relies on participants' interpretation of an ambiguous stimulus. A large body of evidence has shown that individuals high in trait anxiety are biased to interpret ambiguous stimuli in a threatening way [31], [32]–[34]. Thus, when faced with the decision about whether they can trust the person in the image, individuals high in trait anxiety might be more likely to interpret the ambiguity of a neutral expression as threatening. In contrast, approachability judgements are based on how an individual anticipates that he or she would behave in a specific situation, which may be less affected by levels of trait anxiety. In other words, trustworthiness judgements may be determined more by one's cognitions, whereas approachability judgements may be influenced more by how one anticipates that they would behave.

The present results are not consistent with previous research showing that individuals with social anxiety disorder demonstrate abnormal approachability judgements to emotional faces [14]. An obvious explanation for this difference in findings is that social anxiety disorder is characterised by avoidance of social situations, whereas individuals who are high in trait anxiety are not necessary socially avoidant. A further important difference pertains to the fact that the current study comprised neutral faces, whereas Campbell et al. [14] observed a tendency for socially anxious individuals to judge happy faces more negatively than healthy controls. This suggests that social anxiety disorder may be characterised by a particular tendency to interpret positive emotion in a negative manner. Future studies contrasting social anxiety and trait anxiety may be able to shed further light on the nature of discrepancies between the relationship between these distinct types of anxiety and the social judgements of trustworthiness and approachability assigned to neutral and emotional faces.

While there has been considerable attention towards the cognitive and neural bases of trustworthiness judgements [16], the influence of individual differences on these judgements has been neglected. The current study is the first to demonstrate that individuals with high levels of trait anxiety judge others as less trustworthy. Not only do these findings contribute to our understanding of the factors that influence judgements of trustworthiness but they also add a growing body of research demonstrating abnormal cognitive responses to faces in individuals with heightened levels of anxiety [11], [12]. This illustrates another domain in which elevated levels of trait anxiety can bias one's evaluations and subsequent social interactions with others. The observed relationship between trait anxiety and trustworthiness judgements illustrates the potential importance of controlling for trait anxiety levels when assessing trustworthiness judgements. This is particularly important for studies involving special populations who often display atypical levels of trait anxiety [17], [18], [19]. For instance, the observation of a significant difference in trustworthiness judgements between autistic individuals and controls previously observed [5] could have emerged as a consequence of a difference between the trait anxiety levels of the autistic and control group.

It is important to recognise that the correlational nature of this research precludes us from concluding that trait anxiety directly affects judgements of trustworthiness. Future research exploring the development of anxiety and social judgements in longitudinal studies may be of value in determining causality. A further limitation evident in this research pertains to the lab-based nature of this research. Future research assessing these social judgements in real life scenarios may assist in extending the generalisability of these findings and demonstrating their ecological validity.

In conclusion, we have demonstrated that individuals with higher levels of trait anxiety perceive affectively neutral faces as less trustworthy than those with lower levels of trait anxiety. In contrast, trait anxiety levels do not appear to be significantly associated with the perception of approachability. These results demonstrate an important difference in terms of the factors that determine these two social judgements. The finding that trait anxiety is associated with trustworthiness judgements illustrates the importance of assessing trait anxiety in future studies assessing trustworthiness judgements, particularly those studies involving clinical populations who report abnormal levels of anxiety.
