# Correction: The Relationship between Anxiety and the Social Judgements of Approachability And Trustworthiness

**DOI:** 10.1371/annotation/5ef1d1b9-87d8-4946-a0ef-25d65d30bb19

**Published:** 2013-10-07

**Authors:** Megan L. Willis, Helen F. Dodd, Romina Palermo

As a result of issues in the production process, there were errors in the first two two affiliations. Correct versions of these affiliations are available below.

School of Psychology, Australian Catholic University, Sydney, New South Wales, Australia

Department of Cognitive Science, Macquarie University, Sydney, New South Wales, Australia 

